# Hyperspectral remote sensing for tobacco quality estimation, yield prediction, and stress detection: A review of applications and methods

**DOI:** 10.3389/fpls.2023.1073346

**Published:** 2023-03-08

**Authors:** Mingzheng Zhang, Tian’en Chen, Xiaohe Gu, Dong Chen, Cong Wang, Wenbiao Wu, Qingzhen Zhu, Chunjiang Zhao

**Affiliations:** ^1^ School of Agricultural Engineering, Jiangsu University, Zhenjiang, Jiangsu, China; ^2^ Technology Center, Nongxin Smart Agricultural Research Institute, Nanjing, Jiangsu, China; ^3^ Information Engineering Department, National Engineering Research Center for Information Technology in Agriculture, Beijing, China; ^4^ Research Center of Information Technology, Beijing Academy of Agriculture and Forestry Science, Beijing, China

**Keywords:** tobacco, hyperspectral remote sensing, quality estimation, yield prediction, stress detection, vegetation index, machine learning

## Abstract

Tobacco is an important economic crop and the main raw material of cigarette products. Nowadays, with the increasing consumer demand for high-quality cigarettes, the requirements for their main raw materials are also varying. In general, tobacco quality is primarily determined by the exterior quality, inherent quality, chemical compositions, and physical properties. All these aspects are formed during the growing season and are vulnerable to many environmental factors, such as climate, geography, irrigation, fertilization, diseases and pests, etc. Therefore, there is a great demand for tobacco growth monitoring and near real-time quality evaluation. Herein, hyperspectral remote sensing (HRS) is increasingly being considered as a cost-effective alternative to traditional destructive field sampling methods and laboratory trials to determine various agronomic parameters of tobacco with the assistance of diverse hyperspectral vegetation indices and machine learning algorithms. In light of this, we conduct a comprehensive review of the HRS applications in tobacco production management. In this review, we briefly sketch the principles of HRS and commonly used data acquisition system platforms. We detail the specific applications and methodologies for tobacco quality estimation, yield prediction, and stress detection. Finally, we discuss the major challenges and future opportunities for potential application prospects. We hope that this review could provide interested researchers, practitioners, or readers with a basic understanding of current HRS applications in tobacco production management, and give some guidelines for practical works.

## 1 Introduction

**Figure 1:**
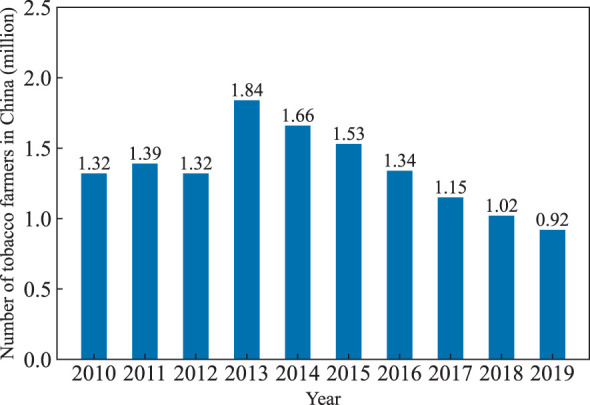
Number of tobacco farmers in China during 2010-2019 (million).

As the primary raw material for a variety of cigarette products, tobacco is one of the most important economic crops, both in China and around the world. China grows nearly one-third of the world’s tobacco crop (Hu et al., 2010). The relevant industries provide the governments with substantial fiscal revenue. The enormous economic benefits are inextricably linked to the meticulous field management of countless practitioners. However, in recent years, the tobacco industry begins to face bottlenecks in development. On the one hand, with the gradual increase in awareness of tobacco risks, people’s attitudes toward tobacco consumption have changed. Tobacco products are no longer seen as ordinary commodities, but as harmful ones. A number of consumers are seeking high-quality, less harmful products. On the other hand, as shown in **Figure 1**, from 2013 to 2019, the number of tobacco farmers declines from 1.84 million to 0.92 million, a reduction of nearly 50% ^1^, leading to unstable yields and unsustainable development (Jia Kang, 2020). The reasons are mainly due to the tobacco planting is a labor-intensive industry with high labor intensity and needs to purchase roasting facilities. The cost benefit ratio of tobacco is lower than other crops (e.g., soybean, corn, and peanuts). Moreover, current tobacco field management methods still largely rely on the experience of tobacco farmers. Take fertilization as an example: when to fertilize, which areas to fertilize, and how much to fertilize are all determined by farmers’ observation. The advantages of this empirical method are simple and fast. Because it doesn’t require any assistance from the instruments. However, it requires farmers or practitioners to be able to make a rapid and accurate diagnosis of tobacco growth and quality, which is not easy in practice. This qualitative approach not only doesn’t reduce the field management costs, but also affects the accurate assessment of tobacco growth status and quality. Besides the empirical method, laboratory testing is another commonly used tobacco quality diagnosis method (Deng et al., 2020). It can measure various agronomic parameters quantitatively such as leaf nitrogen, chlorophyll, water, and nicotine content. This approach can accurately obtain information of various components in tobacco leaves. However, it costs more detection time and expenses, requires considerable professional knowledge, which is rarely used in the actual production (Deng et al., 2020).

In light of this, a concept of “precision tobacco agriculture (PTA)” was born. It inherited from the concept of precision agriculture (PA) or site-specific management (SSM) (Bachmaier and Gandorfer, 2009; Carrow et al., 2010). In our view, PTA is a cost-effective method to address above bottlenecks and to achieve sustainable development (Chang et al., 2014), and the specific applications of PTA should including growth and quality estimation, yield prediction, and stress detection (e.g., diseases, pests, or heavy metals). In China, the first work on tobacco management zones was carried out in 2009. And the results showed that soil nutrients were similar within the management zones, which provided an information basis for SSM in tobacco fields (Xin-Zhong et al., 2009). In this case, the field information acquisition was the first step in PTA applications. However, the traditional destructive field sampling methods and laboratory measurement are generally labor-intensive and time-consuming. Therefore, there is a great demand for a method that can accurately and quickly obtain the field information on tobacco growth and quality during the growing seasons.

**Figure 2:**
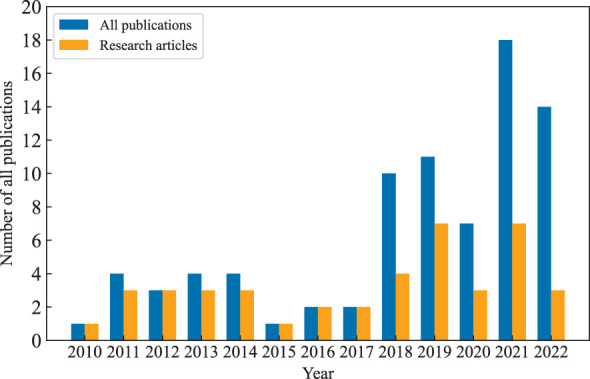
Search results with the topic of “tobacco” and “hyperspectral” in Web of Science from 2010 to 2022.

Fortunately, HRS technology, with its contactless observation, high spectral resolution, and flexibility, is gradually becoming recognized as a suitable alternative to traditional field sampling methods to obtain crop information (Park and Lu, 2015; Ma et al., 2022). In the field of agricultural, the most important ability of HRS is that it can obtain sufficient hyperspectral reflectance data of crops with a non-destructive mean, and with the assistance of various regression modeling algorithms, the relationship between reflectance data and various crop agronomic traits (e.g., leaf nitrogen, chlorophyll, water content, etc.) can be inferred quantitatively (Weiss et al., 2020; Jiang et al., 2022). This process is known as “spectral inversion”. Furthermore, with the development of UAV system platforms and lightweight hyperspectral imaging sensors, the inversion missions of those large-scale or scattered farmlands will become easier and faster (Johansen et al., 2019; Liao et al., 2020). The UAV-borne HRS has demonstrated a bright application prospect (Aasen et al., 2015; Zhong et al., 2018). As for HRS applications for PTA applications, it has also made great improvements (Long et al., 2019; Zhu et al., 2020). According to our survey, studies on tobacco are increasing yearly. **Figure 2** shows the number of related publications from 2010 to 2022. The data are from the “Web of Science” (https://www.webofscience.com) website with the topics “tobacco” and “hyperspectral”. We can find a gradual increase in the number of publications on tobacco. However, a large portion of them are patents. There are not many research articles. This is also one of the reasons why we drafted this review. We hope that interested researchers gain some insight into the latest advances in scientific research of HRS for PTA applications from our collection and summary.

In this review, we comprehensively retrieved the research of HRS in PTA to provide readers or researchers with an enhanced perceptiveness. The cited references mainly come from the Web of Science, IEEE Xplore (https://ieeexplore.ieee.org), and Google Scholar (https://scholar.google.com) websites. A few form the CNKI ^2^ (https://www.cnki.net) website. The literature types include academic journals, international conferences, professional books, and dissertations. Retrieval keywords consist of “hyperspectral remote sensing”, “agricultural remote sensing”, “tobacco remote sensing”, “UAV hyperspectral & tobacco”, and the combinations of them. To ensure the timeliness of references, we tend to adopt literatures with relatively recent publication dates. Thus, references cited in this review were mainly published from 2010 to 2022. Furthermore, according to our retrieval results, the existing reviews related HRS and agriculture applications cover various aspects: UAV-borne HRS (Xiang et al., 2019), hyperspectral imaging technologies (Adão et al., 2017; Mahlein et al., 2018), precision agricultural applications (Bégué et al., 2018; Latif, 2018), leaf area index (LAI) (Ke et al., 2016), crop yield prediction and nitrogen status assessment (Chlingaryan et al., 2018; Fu et al., 2021), wheat grain protein (Ma et al., 2022), etc. The rest of this article is organized as follows: in section 2, we introduce the principles and workflow of HRS applications for PTA; in section 3, we compare three commonly used hyperspectral data acquisition system platforms; the details of specific applications and methodologies are presented in section 4; the discussion of issues and recommendations is arranged in section 5; the conclusion is in section 6. We hope that new readers and researchers will have a holistic view according to our presentation.

## 2 Principles and workflow of HRS for PTA

**Figure 3:**
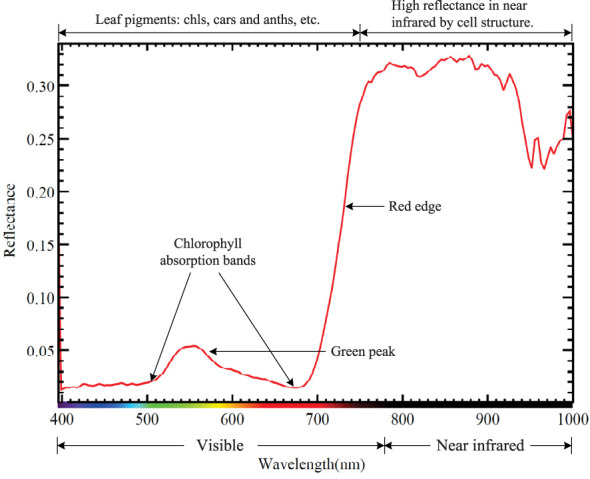
Major absorption and reflection features and locations of tobacco leaves.

Minerals on earth usually have unique diagnostic spectrum reflectance signatures (Vane and Goetz, 1993). Green plants, or plant ecosystems, are composed of the same compounds, which also have numerous unique diagnostic absorption features in the solar reflected spectrum from 400 to 2500nm. To give readers a visualized understanding of this unique feature. **Figure 3** shows a typical reflectance curve of tobacco leaves containing several absorption and reflection features (400-1000 nm) caused by various biological parameters such as chlorophyll, water, and protein. This characteristic allows us to determine the physical, chemical, and biological compositions of plants with the help of remote sensing technologies, which are built on spectral radiometry theory (Borengasser et al., 2007).

**Figure 4:**
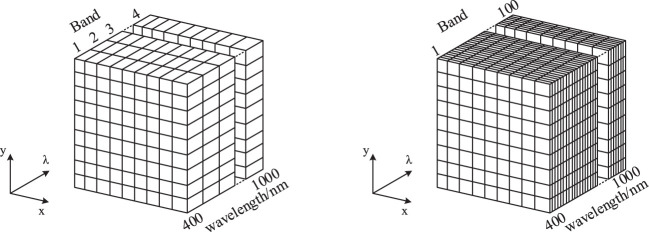
Comparison of MRS (left) and HRS (right) data, *x*, *y* indicate the spatial domain, λ indicates the spectral domain.

As the frontier technology of the current remote sensing field, hyperspectral imaging technologies can obtain sufficient spectra information of ground objects from each pixel in an image of a scene. Hyperspectral reflectance data also have been verified to be more efficient in crop phenotypic traits estimation (Wang et al., 2018; Angel and McCabe, 2022), as well as target classification and precision agriculture (Teke et al., 2013; Zhao et al., 2018). Compared to traditional multispectral remote sensing (MRS), the main differences between HRS and MRS include two aspects. The first is HRS imaging sensors can obtain image data in several hundred narrow and contiguous spectral bands, while the MRS sensors can only measure image data in a few wide and discrete spectral bands. As shown in **Figure 4**, the wavelength range is from 400 to 1000 nm. The MRS has four discrete bands, usually including red, green, blue, and near-infrared bands, whereas the HRS has 100 contiguous bands. The second is HRS data can be used to extract the spectral features of most natural materials, which MRS data cannot do. HRS images contain much more spectral information than MRS. So, HRS has a greater potential for detecting differences among materials on the earth’s 120 surface (Pu, 2017).

**Figure 5:**
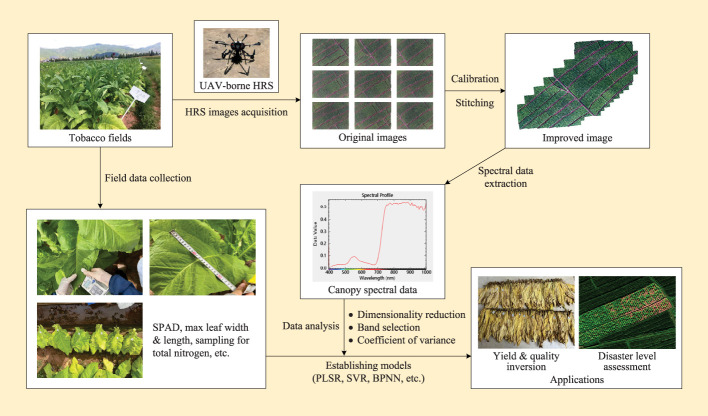
The general workflow of UAV-borne HRS for PTA applications.

**Figure 5** shows the general workflow of HRS applications for tobacco quality estimation, yield prediction, and disaster level assessment. The first is UAV-borne hyperspectral image data acquisition. The complete improved image can be obtained by stitching and alignment the original images. Radiometric correction and geometric calibration are also necessary to reduce noise interference, improve reflectance precision and radiometric accuracy. These operations can convert the original images into the hyperspectral reflectance data of the whole tobacco fields. Radiometric correction is essential for correcting systematic error and radiation distortion (Watts et al., 2012). Considering the atmospheric gases and aerosols absorption during the image collection, methods based on the radiation transmission theory have been widely used for radiation correction, such as MODTRAN (Berk et al., 2014), 6S (Hu et al., 2013), and FLAASH (Vibhute et al., 2015). Moreover, due to the effects of hyperspectral sensors, system platforms, and terrains in data acquisition. The generated image pixels are squeezed, stretched, distorted, and offset with respect to the actual position of planting areas. Thus, geometric correction is necessary too. In practice, both radiometric and geometric corrections are well-established techniques that can be processed directly in professional software (e.g. ENVI, ERDAS, and IDRISI).

The obtained hyperspectral image contains sufficient spectra information of tobacco leaves from each pixel in an image of a scene. However, not all spectral bands are sensitive to the observed indicators. The sensitivity of different bands is varied. Besides, hyperspectral data also have a high dimensionality and high similarity of adjacent bands. So, it is necessary to perform dimensionality reduction and denoising. In order to select the most sensitive spectral bands, various data transformation or feature extraction algorithms are applied, such as the principal component analysis (PCA), the successive projection algorithm (SPA), the elastic net (EN) algorithm, and fuzzy clustering (Koonsanit et al., 2012; Cohen et al., 2013; Liu and Li, 2017; Zhang et al., 2017). The details of those commonly used data dimensionality reduction methods are described in the review Sun and Du (2019).

After performing hyperspectral dimensionality reduction, spectral inversion is conducted to extract information from hyperspectral images for various data mining tasks. Here, inversion modeling plays an important role in quantitative estimation. It bridges the gap between hyperspectral reflectance data and agronomic traits of tobacco. In general, most of the inversion methods can be divided into empirical models and mechanistic approaches, or a combination of them Weiss et al. (2020). The empirical models mainly rely on data collection and statistics, also known as “regressions”, such as partial least squares regression (PLSR) (Dong et al., 2015), support vector machine (SVM) (Mountrakis et al., 2011), random forest (RF) (Johansen et al., 2020), neural networks (Yuan et al., 2017), etc. Its main task is to fit the numerical relationship between the measured agronomy traits in practical and spectral features. As for the mechanistic methods, they are mainly based on assumptions and modeling. For example, radiative transfer model (RTM) (Erten et al., 2016), physically-based model (Verrelst et al., 2019), SVM (Rivera et al., 2015), and neural network (Ermida et al., 2017). Furthermore, the deep learning methods of convolutional neural networks (CNN) are very effective for extracting agronomic features from HRS images (Kattenborn et al., 2021). The existing studies demonstrate that CNN can be utilized in various specific problems, such as tree species classification (Sothe et al., 2020), palm tree detection (Freudenberg et al., 2019), mapping plant communities (Wagner et al., 2019), etc. It provides researchers with a more effective HRS image analysis method, and numerous works have demonstrated that CNN outperforms shallow machine learning methods.

## 3 Available HRS data acquisition systems

Data is the most important part of HRS for tobacco agronomic traits analysis. In general, there are two kinds of data that are necessary. One is the hyperspectral reflectance data of tobacco fields. Another is the corresponding agronomic parameter data. The former can be obtained by various hyperspectral data acquisition systems (e.g., handheld spectrometer, UAV-borne, and satellite). The latter is measured by specific instruments and laboratory analysis (e.g., LAI-2500, SPAD-502Plus, and AutoAnalyzer 3), which may take some time to get results. In this section, we have a brief comparison between the handheld spectrometer, UAV-borne, and satellite systems. An intuitive comparison can be found in **Table 1**.

**Table 1:** A comparison between handheld spectrometer, UAV-borne, and satellite-borne HRS platforms.

Platform	Height	Weight	Spatial resolution	Spectrum	Revisit rate	Holding cost
Handheld	1.3m	5.44kg	Millimeter-level	0.35-2.5um	Minutes	Medium
UAV	80m	10.6kg	Centimeter-level	0.4-1um	Hours	High
Satellite	705km	250kg	Meter-level	0.4-14.4um	Days	Huge

The above parameters are referenced to ASD FieldSpec 4, DJ M600-borne GaiaSky-mini3-VN, and MODIS, respectively.

The first is handheld spectrometers (e.g., ASD FieldSpec 4, Specim-IQ). They have a high resolution and signal-to-noise ratio, better intensity accuracy and wavelength accuracy, as well as strong resistance to external interference and excellent instrument stability. They also come with a collection of great calculation tools and can perform some complex calculations, such as derivation, deconvolution, etc. In the agricultural field, due to its small size, lightweight, and convenient carrying. Some field experiments can be carried out and the measurement results can be displayed within seconds, which greatly improves efficiency. Thus, they are widely adopted for crop agronomic traits monitoring (Jia et al., 2013b; Liang et al., 2018; Cao et al., 2021). The shortcoming is that they take a lot of time to collect data due to the small coverage, especially when dealing with large planting areas.

The second is the UAV-borne HRS system platforms (e.g., V185G, GaiaSky-mini3-VN). UAV platforms are more flexible, especially in terms of revisit frequency. They can perform observation tasks in a specific area at any time, as long as the meteorological conditions are favorable (e.g., low wind speed, clear sky, and cloudless), which increases the efficiency of hyperspectral image acquisition. The application of UAV platforms makes it possible to obtain and analyze tobacco plants quickly at the canopy level (Inoue et al., 2012; Zhu et al., 2020; Liu et al., 2021). In addition, with the improvement in load capacity and battery endurance, there is also significant performance in face of large-scale regional observation tasks (Li et al., 2022). Compared to handheld spectrometers, UAV platforms save a lot of manual work and time; and compared to satellite platforms, UAV platforms are relatively accurate and convenient observation tools. The working height of UAV-borne HRS is usually 100 m. Thus the spatial resolution of UAV imagery is higher than satellite but lower than handheld. There are also some limitations of the UAV itself, such as flight duration, flight stability, and the maximum load, all of which still need to be improved.

The third is satellite-based hyperspectral data observation system platforms (e.g., GF-5, EO-1 Hyperion, and MODIS^3^). All of them have a greater swath width and larger spatial coverage. It makes them have a significant performance in face of large-area observation tasks (Chaurasia et al., 2006; Wang et al., 2021). But their spatial and temporal resolutions are relatively low. The working height is usually several hundred kilometers and the revisiting cycle often takes a few days. Because of the huge launch and maintenance cost, most of the satellite system platforms are supported by governments or large business organizations (e.g., CNSA^4^, NASA^5^, and Space X). However, the public can access some satellite data for free or by paying some fees (e.g., Landsat, Sentinel, and Gaofen). It should be noted that the quality of satellite hyperspectral images is highly susceptible to environmental factors such as cloud cover, rainy weather, and clutter reflections (Mulla, 2013). So, It may be difficult for the public to collect high-quality satellite hyperspectral images focused on the specific area and timings (Zhong et al., 2018).

## 4 Applications and methods

In this section, the specific studies are introduced from three aspects: quality estimation, yield prediction, and stress detection.

### 4.1 Quality estimation

Tobacco quality is a holistic and dynamic concept, high-quality tobacco evolves over time, geography as well as consumers’ desires. In general, tobacco quality mainly includes four aspects:

- Exterior quality: quality indicators that can be judged by human senses, including leaf color, length & width, structure, chrominance, completeness, etc.
- Inherent quality: the aroma and eating flavor when smoking, completely dependent on human feelings.
- Chemical compositions: usually measured in the laboratory, mainly including total nitrogen, chlorophyll, total sugar, nicotine, protein, starch, etc.
- Physical properties: flammability, absorbent, weight per unit area, electrical conductivity, etc.

The existing researches on tobacco quality estimation are decentralized, and the studies mainly focus on chemical compositions and exterior quality, rarely involving inherent quality and physical properties (not the forte of HRS technology). However, the inherent quality can be inferred by chemical compositions (Shen et al., 2017).

#### 4.1.1 Chemical compositions

##### 4.1.1.1 Nitrogen

Nitrogen is the most important nutrient for tobacco growth. Over-and-under-application of nitrogen fertilizers not only limits tobacco productivity but also leads to a negative impact on quality. The tobacco plants absorb the most nitrogen after 40 days of transplanting. An excessive supply of nitrogen fertilizer will result in the leaves being larger than normal, delaying tobacco maturity. Insufficient nitrogen will also lead to a delay in ripening, leaves becoming brown, and declining quality (Li, 2006). Moreover, low leaf nitrogen content (LNC) makes it taste bland, and high LNC will lead to a pungent smell (Shen et al., 2017). Thus, an accurate estimation of nitrogen status is essential to determine the final quality and total yield, improve the use efficiency of nitrogen fertilizer, and reduce environmental pollution (Li et al., 2019).

Jia et al. (2013b) extracted the central band that is sensitive to tobacco LNC based on the coefficient of determination (*R* ^2^) of the linear regression model using the specific ratio vegetation index (SR) and normalized difference vegetation index (NDVI) as independent variables. The optimum band combination was *R*_590_/*R*_1980_ for SR, and (*R*_1970_-*R*_650_)/(*R*_1970_+*R*_650_) for NDVI. They selected 20 SR and 20 NDVI band combinations with the higher *R* ^2^ as the independent variables of stepwise multiple linear regression (SMLR) and error back propagation neural network (BPNN) models to inverse the tobacco LNC. The experiment results showed that the BPNN model achieved the best performance with *R* ^2^ was 0.91 and the root mean square error (RMSE) was 0.09. The *R* ^2^ and RMSE of the SMLR model were 0.86 and 0.60, respectively. Liang et al. (2014) investigated the relationship between spectral features of tobacco cultivars and their nitrogen use. A ^15^N tracer pot experiment was conducted with four tobacco cultivars under different nitrogen use efficiency. The authors configured two nitrogen levels, N1 (1.0 g/pot) and N2 (3.0 g/pot), and utilized three VIs (i.e., ratio vegetation index (RVI), difference vegetation index (DVI), and NDVI) to evaluate the nitrogen use efficiency.

##### 4.1.1.2 Phosphorus

Phosphorus is an essential mineral element required for tobacco photosynthesis and respiration. Li et al. (2014) generated a visual reporting system to monitor the dynamic changes of phosphorus concentration by expressing a purple gene extracted from cauliflower. The authors selected wild-type and transgenic tobacco plants as the experiment targets and studied their correlation between leaf phosphorus concentration and the hyperspectral reflectance at 554 nm. The results showed that the *R* ^2^ of transgenic tobacco leaves was 0.96, and the *R* ^2^ of wild-type leaves was only 0.45.

##### 4.1.1.3 Potassium

Potassium is also an essential mineral element that can increase the intensity of photosynthesis. In general, the higher content of potassium in tobacco leaves, the higher yields and quality will be. Li (2006) studied the quantitative relationship between the leaf potassium concentration and 19 spectral parameters of tobacco. The modeling method was exponential fit. According to the fitting results, there were three spectral parameters achieved better performance: pigment-specific simple ratio (PSSRa), optimized soil adjusted vegetation index (OSAVI), and NDVI (670, 780 nm), the corresponding *R* ^2^ were 0.929, 0.928, and 0.927, respectively. Junying et al. (2020a) proposed a method to predict tobacco *K*_2_ *O* content based on UAV-borne hyperspectral imaging. The model equation was:


(1) $$Y=5.423-486.029\times R_{498.6},$$

where *Y* was the predicted value of tobacco *K*_2_ *O* content, and *R*_498.6_ was the first derivative of the logarithm of original reflectivity at 498.6 nm. The results on test set showed that the RMSE of this model was 0.40, and the absolute value of the mean relative error was 8.04%.

##### 4.1.1.4 Chlorophyll

Chlorophyll is an important indicator in the process of plant growth, including photosynthetic rate, nutritional status, and maturity (Peng and Gitelson, 2012). Especially for tobacco, a broad-leaf crop with leaves harvested, leaf chlorophyll content plays an important role in growth and quality. Guo et al. (2019) investigated the relationship between leaf chlorophyll content (LCC) and various tobacco canopy hyperspectral parameters, including 9 parameters based on red edge position, 3 parameters based on red edge area, and 6 parameters based on VIs. Among them, 7 parameters with high significant level were taken as the independent variables of six regression functions to build inversion models (i.e., linear, exponential, parabolic, power, logarithm, and cubic regression models). Thus, there were 42 inversion results in total. The combination of (*SD_r_-SD_y_*)/(*SD_r_+SD_y_*) ^6^ and linear regression obtained the best performance with *R* ^2^ = 0.948, RMSE=0.127 mg/g, and relative error (RE)=9.31%. Jia et al. (2020) conducted an spectral inversion of tobacco chlorophyll-*a* content under different light qualities. The leaf spectral reflectance data was collected by an ASD field spectrometer. Linear regression and BPNN models were applied to predict leaf chlorophyll-*a* content. The results demonstrated that BPNN has the most reliable performance with *R* ^2^ = 0.86 and RMSE=0.05. A similar study can also be found in Dongyun et al. (2015). Roughly the same parameters were used to estimate the LCC of tobacco leaves infected by the mosaic virus. The best correlation was achieved for the combination of (*SD_r_-SD_y_*)/(*SD_r_+SD_y_*) and *SD_r_*/*SD_b_* under the stepwise regression model (*R* ^2^ = 0.885).

##### 4.1.1.5 Total sugar

Total sugar is an important biochemical indicator reflecting the quality of tobacco leaves. It has a balanced effect on the taste of tobacco products. Junying et al. (2020b) proposed a method to predict the total sugar content based on UAV-borne hyperspectral imaging. The model was built by combining the spectral characteristics and the measured total sugar values. The function formula was:


(2) $$Y=24.74-3384.014\times R_{863.59}-1786.102\times R_{414.7}-2741.762\times R_{469.29},$$

where *Y* was the predicted content of total sugar, *R*_863.59_, *R*_414.7_, and *R*_469.29_ denoted the first derivative of the logarithm of the original spectral reflectance at 863.59, 414.7, and 469.29 nm, respectively. According to the sample test results, the RMSE of this model was 1.84, and the absolute value of the mean relative error was 8.82%. Soares et al. (2019) developed an inline simultaneous analytical method to quantify the leaf sugar content using near-infrared hyperspectral imaging. The inversion model was established offline using partial least square regression (PLSR). The *R* ^2^ and RMSE were 0.778 and 2.28, respectively.

##### 4.1.1.6 Alkaloid

Nicotine is the main alkaloid in tobacco and is the primary factor in the commercial value of tobacco Henry et al. (2019). Moreover, nicotine is also the foremost chemical that influences tobacco quality. The leaf nicotine content is a key indicator for estimating the quality of fresh tobacco leaves (Dou et al., 2016). In order to quantitatively determine the relationship between leaf nicotine content and spectral reflectance, Jia et al. (2013a) explored the specific bands that can be utilized to detect nicotine. The SMLR and BPNN were applied to establish the inversion model between hyperspectral reflectance and leaf nicotine content. The experiment results showed that BPNN had the most significant performance with *R* ^2^ = 0.968 and RMSE=0.109. Soares et al. (2019) developed an inline simultaneous analytical method to quantify nicotine content using near-infrared hyperspectral imaging. They used PLSR and achieved a result of *R* ^2^ = 0.798 and RMSE=0.447. Dou et al. (2016) evaluated the relationship between 11 spectral parameters and leaf nicotine content. The first-order derivative of reflectance data was calculated to perform a standardized analysis. Furthermore, five methods (e.g., linear, power, logarithmic, exponential, and negative exponential) were utilized to fit the values. The statistical analysis showed that the combination of power function and (*SD_r_-SD_y_*)/(*SD_r_+SD_y_*) obtained the best results with *R* ^2^ = 0.8112, RMSE=0.2272, and relative error (RE)= 14.42%. Divyanth et al. (2022) applied hyperspectral and four machine learning algorithms to predict tobacco nicotine content. The average spectra of region of interest (ROI) were used to establish the inversion model based on PLSR, RF, support vector regression (SVR), and PLSRâ€”variable importance in projection (PLSRâ€”VIP). The models were evaluated using leave-one-out cross-validation and on 15% test set. The results showed that the PLSR (*R* ^2^ = 0.93, RMSE=0.21%) outperformed SVR (*R* ^2^ = 0.89, RMSE=0.36%), RF (*R* ^2^ = 0.90, RMSE=0.35%), and PLSR-VIP (*R* ^2^ = 0.91, RMSE=0.30%).

##### 4.1.1.7 Moisture

Leaf moisture content is an important index for tobacco cultivation and precision field management. Sun et al. (2016) proposed a fast and non-destructive way to evaluate the leaf moisture content of tobacco leaves. Mahalanobis distance coupled with Monte Carlo cross-validation (MCCV) was applied to eliminate outlier samples. Savitzky-golay smoothing (SG), roughness penalty smoothing (RPS), kernel smoothing (KS), and median smoothing (MS) were applied to preprocess the raw data. Then SPA and MLR were used to select crucial bands and build the inversion model, respectively. The results showed that the best model was MD-MCCV-MS (*R* ^2^ = 0.9132, RMSE=0.1162).

#### 4.1.2 Exterior quality

##### 4.1.2.1 Leaf area index

Leaf area index (LAI) is one of the most essential exterior parameters of tobacco. It reflects the tobacco canopy structure and growth status. Two external quality indicators, max leaf width & length, are necessary to determine LAI. So, we classified LAI into external quality. The relevant formula is:


(3) $$LAI=k\varrho \frac{\sum_{j=1}^{m}\sum_{i=1}^{n}\left(L_{ij}\times W_{ij}\right)}{m},$$

where *k* is a constant with a value of 0.6345, ϱ is the planting density, *L_ij_* is the value of leaf length, *W_ij_* is the value leaf width, *m* is the number of measured plants, and *n* is the number of leaves of each plant, respectively.

Chaurasia et al. (2006) estimated the field-scale LAI of tobacco using MRS data from a satellite platform. The ground LAI data were measured by LAI-2000 (LICOR Inc., Nebraska) canopy analyzer. Two regression models (exponential and power functions) were conducted between the measured ground LAI and three vegetation indices (SR, NDVI, SAVI). The power model performed better than the exponential model for LAI estimation (NDVI: *R* ^2^ = 0.62). This work demonstrated the feasibility of satellite MRS data for field-scale LAI estimation, although the correlation is not high. ZhengYang et al. (2011) assessed and compared the performance of some hyperspectral models in terms of their prediction capability of tobacco LAI. The hyperspectral data were collected in different water and nitrogen conditions by handheld spectrometer. Four vegetation indices, NDVI, RVI, modified soil-adjusted vegetation index (MSAVI), and modified second triangular vegetation index (MTVI2). The PCA method was applied for hyperspectral data dimensionality reduction, and BPNN was used for LAI inversion. The *R* ^2^ and RMSE of the BPNN model were 0.889 and 0.195, respectively. Qiao et al. (2011) studied the relationship between NDVI and LAI. A linear regression model was built and the *R* ^2^ was 0.568.

##### 4.1.2.2 Tobacco classification

Tobacco classification is an important method for evaluating the grades of tobacco leaves. The determination of tobacco grades directly involves the purchase prices, which is important for farmers, enterprises, and other parties, so the relevant study is of great significance in practice. Current research advances in tobacco classification have focused on scoring tobacco leaves for size, color, structure, chrominance, or completeness using RGB images (Bose et al., 2016; Fan et al., 2018; Lin et al., 2022). Considering that the hyperspectral images contains more spectral features than RGB images. In this case, we can establish a relationship between the chemical compositions and exterior qualities according to the hyperspectral reflectance data (Liu and Shi, 2020). Thus, the classification accuracy can be greatly improved. And the feasibility has been proven in studies on 366 the classification of tobacco leaves health grades.

Zhu et al. (2017) used three machine learning algorithms to achieve early detection of tobacco mosaic virus *via* hyperspectral images. Herein, the SPA method was adopted to select the effective wavelengths to reduce the redundant spectral information. The RF, SVM, and BPNN were applied to guarantee the detection accuracy and obtain more valuable features. The experiment results showed that the overall accuracy of the train set and test set varied between 84.17-100.00% and 75.00-98.33%, respectively. The study in Gu et al. (2019) attested to the applicability of HRS imaging technology in the detection of tobacco tomato spotted wilt virus (TSWV) infection. The authors adopted three wavelengthÆ’ selection methods, SPA, boosted regression tree (BRT), and genetic algorithm (GA), and four machine learning algorithms, BRT, SVM, RF, and classification and regression tree (CART), to analyze the spectral characteristics of normal and diseased leaves in the range of 400-1000 nm. The results showed that the reflectance curve of healthy leaves was significantly higher than diseased leaves after 5 days of infection. The overall classification accuracy reached 95.8% under the SPA-BRT model. Sahu and Dante (2018) investigate the potential of HRS imaging for cured tobacco classification. A multivariate calibration model was developed using end-member extraction and linear discriminant analysis (LDA). Mahalanobis distance was used to show the differences between different tobacco grades. The classification accuracy can reach 93%.

##### 4.1.2.3 3D modeling

Considering the complex geometry of plants and their interplay with the illumination scenario highly affects spectral information acquisition. Behmann et al. (2015) proposed a 3D modeling method combined hyperspectral images and 3D point clouds. The authors used tobacco leaves as an example and analyzed the effects of plant geometry on NDVI. The geometry sensor with different elevation angles resulted in different NDVI values. The low NDVI values on the horizontal parts were caused by the specular reflection, which was independent from leaf chlorophyll content.

### 4.2 Yield prediction

Tobacco yield predictions are important to stabilize tobacco prices in the marketplace and policy making. The producers need to monitor crop growth and development, an accurate early production forecast is as relevant for farmers as it is for the entire tobacco industry. Svotwa et al. (2013) reviewed the applications of remote sensing in crop area assessment and yield prediction, some recommendations were given for tobacco such as the Garvin model, feasible VIs, etc.

As soil nutrients and fertilizer application play a significant role in tobacco growth and yield. Chang et al. (2014) investigated the potential of NDVI for management zone delineation to build fertilizer applications in tobacco-planted fields. The yield mapping was built through SMLR analysis to find the key yield-limiting factors of soil components and NDVI. The value of NDVI was collected by the GreenSeeker handheld spectrometers. According to the results, the soil organic matter, active phosphorus, and available nitrogen were the main limiting factors for tobacco growth. The results also showed that the value of NDVI_60 (60 days after transplanting) had a relatively high correlation with yield. Falcioni et al. (2022) proposed a rapid quantification method to estimate biomass production using HRS with visible, near-infrared (NIR), and shortwave spectroscopy (SWIR). PCA and PLSR algorithms were used to extract the key wavelengths and built the prediction model of tobacco yield, respectively. The results showed that the most important wavelengths were well distributed into 400 (violet) 440 (blue), 550 (green), 670 (red), 700-750 (red edge), 1330 (NIR), 1450 (SWIR), 1940 (SWIR), and 2200 (SWIR) nm operating ranges of the spectrum. The established model also had an excellent prediction capacity for yield with *R* ^2^ = 0.85 and RMSE=0.93.

Besides the soil nutrients and fertilizer application, photosynthetic capacity is also a major factor affecting crop yield. Increasing photosynthetic capacity remains probably the best strategy for improving crop yields (Ort et al., 2015). Herein, the maximum Rubisco carboxylation (*V_c,max_*) and maximum electron transport rate (*J_max_*) are generally used as indicators to assess photosynthetic capacity. And hyperspectral techniques coupled with machine learning methods are effective in quantifying these parameters (Meacham-Hensold et al., 2019).

Three different methods used the PLSR model with inputs of hyperspectral reflectance (400-900 nm), VIs (SR, modified normalized difference index (mND), and structure insensitive pigment index (SIPI)), and RTM-derived (PROCOSINE model) crop traits, were synthesized and compared with their ability to reveal photosynthetic differences across tobacco species (Fu et al., 2020). The results showed that PLSR with inputs of hyperspectral reflectance and VIs achieved an *R* ^2^ of ∼0.8 for predicting *V_c,max_* and *J_max_*, higher than the *R* ^2^ of ∼0.6 obtained by PLSR of PROCOSINE model. However, the performance of the PLSR model varies significantly across species, regions, and growth environments. To alleviate this bottleneck, Fu et al. (2019) developed a novel ensemble framework that stacked six machine learning algorithms (e.g., artificial neural network (ANN), least absolute shrinkage and selection operator (LASSO), Gaussian process (GP), SVM, RF, and PLSR) to estimate *V_c,max_* and *J_max_*. The ensemble framework was established based on leaf reflectance spectra in the range of 400-2500 nm and six tobacco genotypes. According to the results, the mean *R* ^2^ and RMSE of six regression algorithms for predicting *V_c,max_* (*J_max_*) ranged from 0.60 (0.45) to 0.65 (0.56) and 47.1 (40.1) to 54.0 (44.7) μmol *m* ^-2^ *s* ^-1^, respectively. And the stacking regression performed better than any of the individual models with increases in *R* ^2^ of 0.1 (0.08) and decreases in RMSE by 4.1 (6.6) μmol *m* ^-2^ *s* ^-1^.

### 4.3 Stress detection

Stress detection aims to assess various factors that are detrimental to the survival and growth of tobacco plants, usually caused by infection and competition, such as disease, pests, weeds, heavy metal damage, etc. All of these are the main limiting factors for the final yield and quality of tobacco.

#### 4.3.1 Disease and pest

Hyperspectral imaging technology has been successfully applied for plant disease detection, modeling, and classification (Moghadam et al., 2017). Wang et al. (Wang et al., 2011; Wang et al., 2012) focused on tobacco plants and studied the feasibility of HRS technology to monitor disease and pest stress in natural conditions. The raw hyperspectral data were measured by ASD handheld spectrometers and transformed by the first differential coefficient. The results showed that the wavelengths of 631, 638, 696, 733, and 864 nm were sensitive to severity levels, which provided a theoretical foundation for the application of HRS technology to quantify disease and pest stress levels.

Yusuf and He (2011) investigated the effect of black-shank disease on the spectral characteristics and leaf water content of tobacco. The diseased tobacco plant samples were obtained *via* artificial inoculation. The corresponding reflectance data were collected by the hyperspectral imaging system in the laboratory. PCA and minimum noise fraction (MNF) methods were used to extract pivotal information and remove noise. Plant senescence reflectance index (PSRI) and water band index (WBI) were used to determine the disease level and leaf water content. The results demonstrated the wavelength of 730 and 790 nm were the most useful for discriminating black-shank disease severity levels, with an overall accuracy of 90 to 94%. Krezhova et al. (2014) applied HRS technology to detect TSWV infection at young tobacco plants. The hyperspectral reflectance data were obtained by a handheld spectrometer on the 14th and 20th days after the inoculation. The leaf viral concentration was determined by the serological method, i.e., double antibody sandwich enzyme-linked immunosorbent assay (DAS-ELISA). According to the results, on the 14th day after inoculation, there are no visible changes but the mean spectral reflectance had significant differences between healthy and infected plants at four spectral ranges (green, red, red edge, and NIR regions). And on the 20th day, the infection was deepening and the position of red edge was shifted. The results were consistent with the serological analysis.

Hayes and Reed (2021) conducted a field study using UAV-borne hyperspectral imaging to detect tobacco black-shank disease. In this work, the authors proposed two hyperspectral indices (broad-band index and narrow-band index) to observe the differences in the mean spectral reflectance of symptomatic and asymptomatic tobacco plants. The subspace LDA algorithm was adopted to test the identification ability and obtained an overall accuracy of 85.7%. Hong-Bo et al. (2007) investigated the spectral features of tobacco leaves infected by aphids and had a comparison of different damage levels. The reflectance curve and its first-order derivative curve were selected as the observation indices. And the linear regression model was applied to analyze the leaf chlorophyll content under different aphid damage levels(healthy, light, middle, and severe). The results showed that the values of reflectance curves decreased with increasing damage levels. The descent rate was 12%, 27%, and 52%, respectively. As for the first-order derivative curve, the maximum values of spectral reflectance also decreased as the damage level increased. The maximum values of the derivative were 0.031, 0.022, 0.026, and 0.019, respectively.

#### 4.3.2 Heavy metal

Excess heavy metals in crops will depress normal plant growth and the yield will be harmful if they are loaded into the food chain. Copper ion is an indispensable element for plant growth, but too large concentrations can also impair normal plant growth. Qu and Jiao (2018) investigated the copper ion content of tobacco leaves under copper-stressed conditions from hyperspectral data by inverting a modified RTM (PROSPECT*_cu_*). According to the experiment analysis, the copper ion content had a high sensitivity in the range of 1896-1973 nm. The results showed that the values of *R* ^2^ and RMSE were 0.87 and 0.087, respectively. Yu et al. (2021) aimed to identify the tobacco canopy features that respond to leaves stressed by different concentrations of hydrargyrum (Hg). PCA and the competitive adaptive reweighted sampling (CARS) algorithm were used to reduce the hyperspectral data dimensionality and pick effective wavelengths. Partial least squares discriminant analysis and least-squares SVM (LS-SVM) algorithms were utilized to assess the stress levels of tobacco plants. As a result, the combination of CARS and LS-SVM methods achieved an accuracy of 100%.

#### 4.3.3 Nutrition deficiency

Henry et al. (2023) investigated the spectral differences of tobacco leaves under macronutrient deficiencies. Information entropy and spectral derivatives methods were adopted to identify the efficient wavelengths. PCA and LDA algorithms were used to reduce data dimensionality and classify the symptoms. The results showed that the overall accuracy on young, intermediate, and mature plants was 92%, 82%, and 75%, respectively. The results also showed that the deficiencies of nitrogen, sulfur, and magnesium will affect the classification accuracy to a large extent, but phosphorus and potassium deficiencies had little effect on the results.

## 5 Issues and recommendations

HRS is a non-destructive information acquisition technology about objects from distance. This character is perfect for crop quality estimation, yield prediction, and stress detection. The existing researches show that HRS technology has enormous potential for various agricultural applications. In our view, HRS will be indispensable for digital agriculture and agricultural informatization in the future. Certainly, there are also some problems to be solved, whether the technology itself or the specific applications.

### 5.1 Issues

First of all, the cost of hyperspectral data acquisition is relatively high, no matter the financial or labor cost. Taking the example of a UAV-borne HRS system, the price of UAVs varies by type and function. It may take thousands to tens of thousands of RMB. The carried hyperspectral camera, the price is approximately half a million RMB or more (Feng et al., 2021). Ordinary farmers or research groups rarely have their own UAV-borne HRS devices due to lacking finance and technology support. They usually choose to rent a suite of equipment from the data service providers, and the price is about 50000 RMB per time. As for the ground-based handheld spectrometer, it can provide the highest accuracy of reflectance data with less interference, but it requires operators to traverse the entire field and select suitable samples to collect spectral data. This method is troublesome and time-consuming, especially facing a large-scale area. And its price is about 150000-300000 RMB. Thus, the popularization of agricultural UAV-borne HRS still faces obstacles.

Secondly, external factors will affect the image quality during data collection, such as measuring time, light intensity, solar altitude angle, etc. Due to the limitation of endurance capability, the UAV must complete the mission within a limited time (about 30 minutes). To guarantee image quality, the UAV should keep at a suitable height (about 50-100 m). The obtained hyperspectral images contain the spectral information of all ground objects in the lens, such as crops, soil, roads, and weeds, which may cause noise for targets to distinguish. How to balance the image quality with the flying height, time, spatial resolution, and coverage area still need further investigation.

Thirdly, the ripening and harvesting times of tobacco leaves in different positions are varied (usually 20 days apart). The order of harvesting is bottom, middle, and top, respectively. Some observation tools (e.g., UAVs) can only obtain the canopy reflectance data. Therefore, we can easily find that the canopy spectra are not fully representative of the bottom and middle. In practical research, this problem may lead to large differences between the results obtained by model prediction and the actual values. Besides, the growth status of tobacco seedlings in each period from transplanting to harvesting may also affect the final quality. However, many studies collected plant samples from one stage (e.g., returning seedling stage, root elongation stage, flourishing stage, or maturity stage). So, whether the canopy spectral data in one stage can predict the final tobacco quality is also a question that needs to be verified.

Fourthly, the relevant research about tobacco are decentralized, mainly focusing on one agronomic parameter, and establishing an inversion model based on the corresponding hyperspectral reflectance data. As for the deeper active mechanisms, there are few studies explored. According to the discovery of Li (2006), the potassium ion has significant effects on leaf nitrogen and chlorophyll content. So, the relationship between various biophysical indices is an important basis for spectral inversion. How to exploit these relationships to monitor agronomic parameters of tobacco that are not sensitive to the spectral response is also worth studying.

Finally, the localization and universality of various models. Due to the differences in species, regions, and growing environment, the established inversion models may have some unique geographical features. We named this phenomenon “model localization”. But some researchers prefer universal models. For example, (Feng et al., 2021) hope to construct a universal crop monitoring model based on UAV-borne HRS. The support of existing technologies such as ensemble learning (Fu et al., 2019) and transfer learning (Zhang et al., 2021; Wan et al., 2022) make it possible to build universal models.

### 5.2 Recommendations

The researches mentioned in this review illustrated that the HRS technology was effective for various precision tobacco agriculture scenarios (e.g., quality estimation, yield prediction, and stress detection). However, there are still many challenges to make these studies available to guide the practical production. Here are some recommendations for future studies.

The first is hyperspectral data collection. Recent researches have demonstrated that UAV-borne HRS is a game-changer in precision agriculture, which offers unprecedented spectral, spatial, and temporal resolution (Maes and Steppe, 2019). However, the accuracy of UAV-based data is relatively lower than handheld spectrometers. So, more works with near-ground HRS calibration were needed to strengthen UAV-borne HRS for precision tobacco agriculture applications. Meanwhile, low-cost and high-performance UAVs should be manufactured to make them affordable to more people and to improve the performance of UAV platforms in terms of flight stability, duration and load. In our view, data quality is important, and it relies on high performance sensors, and only a tool that is economical enough will be widely used.

The second is data processing and modeling. Hyperspectral sensors are very sensitive optical components that are highly susceptible to environmental interference. The quality of the obtained data has a significant impact on modeling. And considering the hyperspectral data coupled with field sampling data is indispensable in spectral inversion. Many studies lacked the detection of outliers in field samples. They usually employed one or more algorithms to build simple inversion models and selected the best one which has the highest *R* ^2^ and lowest RMSE. The main work of researchers is to optimize the models and improve their accuracy. We think this is detrimental to the development of the remote sensing community. In future studies, more universal models should be introduced based on some novel technologies such as ensemble learning and transfer learning. Of course, methods to reduce the noise caused by environmental factors should also be proposed.

The third is to pay more attention to multi-parameter and multi-stage models for quality estimation, yield prediction, or stress detection. The existing literature mainly performs inversion or predictive modeling based on a single parameter or growth stage. It’s necessary to investigate the dynamic development of tobacco phenotypic traits at different growth stages. Furthermore, the canopy reflectance spectrum is a comprehensive indicator. It is the result of all factors (internal and external) reacting together. So, the inversion models based on multi-parameter are meaningful to improve the overall accuracy. It can be used as a new research direction in the future.

The fourth is the relationship between the inherent quality and chemical compositions of tobacco. Currently, the inherent quality estimation mainly depends on the feelings of people smoking. However, this method requires evaluators to smoke frequently, which is very harmful to their health. So, we have an idea that the first step is the quantitative inversion of chemical compositions using HRS, and the second step is to establish the quantitative or qualitative relationships between chemical compositions and inherent qualities. The objective is to find the optimal range of each chemical composition corresponding to high-quality tobacco that meets consumers’ demands.

## 6 Conclusions

In this paper, we focused on the application of HRS in precision tobacco agriculture and presented a comprehensive review of related applications and methodologies in terms of quality estimation, yield prediction, and stress detection. Compared to traditional destructive field sampling, laboratory testing, and MRS methods, HRS can provide unprecedented spectral, spatial, and temporal resolution. We compared three commonly used HRS system platforms: handheld, UAV, and satellite. Both of them have benefits, shortcomings, and suitable scenarios. We also depicted a detailed technology roadmap of UAV-borne HRS for precision tobacco agriculture. As for the specific applications, we summarized in three parts: quality estimation, yield prediction, and stress detection. The relevant modeling methods and their performances were also analyzed. In summary, the key issue is how to establish the quantitative inversion models between spectral features and the corresponding observation indices. The commonly used methods for hyperspectral data dimensionality reduction are PCA, SPA, GA, clustering analysis, etc. And inversion models are usually driven by PLSR, PCR, MLR, BPNN, SVM, RF, etc. Several studies used stacking regression. The independent variables of these algorithms are usually full-band spectrum, key-band spectrum, first-order derivative spectrum, and various VIs. Also, how to improve the accuracy and universality of the relevant models is still a challenge that needs to be solved.

## Author contributions

MZ: Conceptualization, Methodology, Formal analysis, Data Curation, Writing - Original Draft, Visualization; T’eC, XG, WW, and CZ: Supervision, Project administration, Funding acquisition; DC, CW, and QZ: Data Curation, Investigation. All authors contributed to the article and approved the submitted version.

## Footnotes

1 Data source: The State Tobacco Monopoly Administration’s annual National Tobacco Work Report, evaluated based on the national total income of tobacco farmers and average income per household.

2 China National Knowledge Infrastructure

3 Moderate Resolution Imaging Spectroradiometer

4 China National Space Administration

5 National Aeronautics and Space Administration

6
*SD_r_*_(_ *_y_*_)_was the sum of the first-order derivatives inside red (yellow) edge.

## References

[B1] Aasen H. Burkart A. Bolten A. Bareth G. (2015). Generating 3d hyperspectral information with lightweight uav snapshot cameras for vegetation monitoring: From camera calibration to quality assurance. ISPRS J. Photogramm. Remote Sens. 108, 245–259. doi: 10.1016/j.isprsjprs.2015.08.002

[B2] Adão T. Hruška J. Pádua L. Bessa J. Peres E. Morais R.. (2017). Hyperspectral imaging: A review on uav-based sensors, data processing and applications for agriculture and forestry. Remote Sens. 9, 1110. doi: 10.3390/rs9111110

[B3] Angel Y. McCabe M. F. (2022). Machine learning strategies for the retrieval of leaf-chlorophyll dynamics: Model choice, sequential versus retraining learning, and hyperspectral predictors. Front. Plant Sci. 13. doi: 10.3389/fpls.2022.722442 PMC8963469 35360313

[B4] Bachmaier M. Gandorfer M. (2009). A conceptual framework for judging the precision agriculture hypothesis with regard to site-specific nitrogen application. Precis. Agric. 10, 95–110. doi: 10.1007/s11119-008-9069-x

[B5] Bégué A. Arvor D. Bellon B. Betbeder J. De Abelleyra D. PD Ferraz R.. (2018). Remote sensing and cropping practices: A review. Remote Sens. 10, 99. doi: 10.3390/rs10010099

[B6] Behmann J. Mahlein A.-K. Paulus S. Kuhlmann H. Oerke E.-C. Plümer L. (2015). “Generation and application of hyperspectral 3d plant models,” in European conference on computer vision. (Zurich, Switzerland: Springer), 117–130. doi: 10.1007/978-3-319-16220-1_9

[B7] Berk A. Conforti P. Kennett R. Perkins T. Hawes F. Van Den Bosch J. (2014). “Modtran^®^ 6: A major upgrade of the modtran^®^ radiative transfer code,” in 2014 6th Workshop on Hyperspectral Image and Signal Processing: Evolution in Remote Sensing (WHISPERS). (Lausanne, Switzerland: IEEE), 1–4. doi: 10.1109/WHISPERS.2014.8077573

[B8] Borengasser M. Hungate W. S. Watkins R. (2007). Hyperspectral remote sensing: principles and applications (Boca Raton, Florida: CRC press). doi: 10.1201/9781420012606

[B9] Bose P. Kasabov N. K. Bruzzone L. Hartono R. N. (2016). Spiking neural networks for crop yield estimation based on spatiotemporal analysis of image time series. IEEE Trans. Geosci. Remote Sens. 54, 6563–6573. doi: 10.1109/TGRS.2016.2586602

[B10] Cao C. Wang T. Gao M. Li Y. Li D. Zhang H. (2021). Hyperspectral inversion of nitrogen content in maize leaves based on different dimensionality reduction algorithms. Comput. Electron. Agric. 190, 106461. doi: 10.1016/j.compag.2021.106461

[B11] Carrow R. N. Krum J. M. Flitcroft I. Cline V. (2010). Precision turfgrass management: Challenges and field applications for mapping turfgrass soil and stress. Precis. Agric. 11, 115–134. doi: 10.1007/s11119-009-9136-y

[B12] Chang D. Zhang J. Zhu L. Ge S.-H. Li P.-Y. Liu G.-S. (2014). Delineation of management zones using an active canopy sensor for a tobacco field. Comput. Electron. Agric. 109, 172–178. doi: 10.1016/j.compag.2014.09.019

[B13] Chaurasia S. Bhattacharya B. K. Dadhwal V. K. Parihar J. S. (2006). Field-scale leaf area index estimation using irs-1d liss-iii data. Int. J. Remote Sens. 27, 637–644. doi: 10.1080/01431160500262620

[B14] Chlingaryan A. Sukkarieh S. Whelan B. (2018). Machine learning approaches for crop yield prediction and nitrogen status estimation in precision agriculture: A review. Comput. Electron. Agric. 151, 61–69. doi: 10.1016/j.compag.2018.05.012

[B15] Cohen S. Cohen Y. Alchanatis V. Levi O. (2013). Combining spectral and spatial information from aerial hyperspectral images for delineating homogenous management zones. Biosyst. Eng. 114, 435–443. doi: 10.1016/j.biosystemseng.2012.09.003

[B16] Deng X. Zhu Z. Yang J. Zheng Z. Huang Z. Yin X.. (2020). Detection of citrus huanglongbing based on multi-input neural network model of uav hyperspectral remote sensing. Remote Sens. 12, 2678. doi: 10.3390/rs12172678

[B17] Divyanth L. Chakraborty S. Li B. Weindorf D. C. Deb P. Gem C. J. (2022). Non-destructive prediction of nicotine content in tobacco using hyperspectral image–derived spectra and machine learning. J. Biosyst. Eng. 47, 106–117. doi: 10.1007/s42853-022-00134-0

[B18] Dong T. Meng J. Shang J. Liu J. Wu B. Huffman T. (2015). Modified vegetation indices for estimating crop fraction of absorbed photosynthetically active radiation. Int. J. Remote Sens. 36, 3097–3113. doi: 10.1080/01431161.2015.1042122

[B19] Dongyun X. Xinju L. Yuqing D. Mingqin L. Yonghua Y. Jijun N.. (2015). Estimation of the chlorophyll contents of tobacco infected by the mosaic virus based on canopy hyperspectral characteristics. JAPS J. Anim. Plant Sci. 25, 158–164.

[B20] Dou Y. Q. Cheng S. Li X. J. Liu Y. Yuan X. L. (2016). Estimation of nicotine content in tobacco leaves based on hyperspectral imaging. Appl. Ecol. Environ. Res. 15, 1419–1426. doi: 10.15666/aeer/1504_14191426

[B21] Ermida S. L. DaCamara C. C. Trigo I. F. Pires A. C. Ghent D. Remedios J. (2017). Modelling directional effects on remotely sensed land surface temperature. Remote Sens. Environ. 190, 56–69. doi: 10.1016/j.rse.2016.12.008

[B22] Erten E. Lopez-Sanchez J. M. Yuzugullu O. Hajnsek I. (2016). Retrieval of agricultural crop height from space: A comparison of sar techniques. Remote Sens. Environ. 187, 130–144. doi: 10.1016/j.rse.2016.10.007

[B23] Falcioni R. Moriwaki T. Antunes W. C. Nanni M. R. (2022). Rapid quantification method for yield, calorimetric energy and chlorophyll a fluorescence parameters in nicotiana tabacum l. using vis-nir-swir hyperspectroscopy. Plants 11, 2406. doi: 10.3390/plants11182406 36145806 PMC9501474

[B24] Fan Z. Lu J. Gong M. Xie H. Goodman E. D. (2018). Automatic tobacco plant detection in uav images *via* deep neural networks. IEEE J. Select. Topics Appl. Earth Obs. Remote Sens. 11, 876–887. doi: 10.1109/JSTARS.2018.2793849

[B25] Feng L. Chen S. Zhang C. Zhang Y. He Y. (2021). A comprehensive review on recent applications of unmanned aerial vehicle remote sensing with various sensors for high-throughput plant phenotyping. Comput. Electron. Agric. 182, 106033. doi: 10.1016/j.compag.2021.106033

[B26] Freudenberg M. Nölke N. Agostini A. Urban K. Wörgötter F. Kleinn C. (2019). Large Scale palm tree detection in high resolution satellite images using u-net. Remote Sens. 11, 312. doi: 10.3390/rs11030312

[B27] Fu P. Meacham-Hensold K. Guan K. Bernacchi C. J. (2019). Hyperspectral leaf reflectance as proxy for photosynthetic capacities: An ensemble approach based on multiple machine learning algorithms. Front. Plant Sci. 10, 730. doi: 10.3389/fpls.2019.00730 31214235 PMC6556518

[B28] Fu P. Meacham-Hensold K. Guan K. Wu J. Bernacchi C. (2020). Estimating photosynthetic traits from reflectance spectra: a synthesis of spectral indices, numerical inversion, and partial least square regression. Plant Cell Environ. 43, 1241–1258. doi: 10.1111/pce.13718 31922609 PMC7385704

[B29] Fu Y. Yang G. Pu R. Li Z. Li H. Xu X.. (2021). An overview of crop nitrogen status assessment using hyperspectral remote sensing: Current status and perspectives. Eur. J. Agron. 124, 126241. doi: 10.1016/j.eja.2021.126241

[B30] Gu Q. Sheng L. Zhang T. Lu Y. Zhang Z. Zheng K.. (2019). Early detection of tomato spotted wilt virus infection in tobacco using the hyperspectral imaging technique and machine learning algorithms. Comput. Electron. Agric. 167, 105066. doi: 10.1016/j.compag.2019.105066

[B31] Guo T. Tan C. Li Q. Cui G. Li H. (2019). Estimating leaf chlorophyll content in tobacco based on various canopy hyperspectral parameters. J. Ambient Intell. Humanized Comput. 10, 3239–3247. doi: 10.1007/s12652-018-1043-5

[B32] Hayes A. Reed T. D. (2021). Hyperspectral reflectance for non-invasive early detection of black shank disease in flue-cured tobacco. J. Spectral Imaging 10, 1–10. doi: 10.1255/jsi.2021.a4

[B33] Henry J. B. Vann M. C. Lewis R. S. (2019). Agronomic practices affecting nicotine concentration in flue-cured tobacco: A review. Agron. J. 111, 3067–3075. doi: 10.2134/agronj2019.04.0268

[B34] Henry J. Veazie P. Furman M. Vann M. Whipker B. (2023). Spectral discrimination of macronutrient deficiencies in greenhouse grown flue-cured tobacco. Plants 12, 280. doi: 10.3390/plants12020280 36678993 PMC9863923

[B35] Hong-Bo Q. Jin-Wei J. Deng-Fa C. Sheng-Li C. Jian-An L. Ji-Sheng M. (2007). Comparison of hyperspectral characteristics in tobacco aphid damage. Chin. Bull. Entomol. 44, 57–61. doi: 10.3969/j.issn.0452-8255.2007.01.014

[B36] Hu Y. Liu L. Liu L. Peng D. Jiao Q. Zhang H. (2013). A landsat-5 atmospheric correction based on modis atmosphere products and 6s model. IEEE J. Select. Topics Appl. Earth Obs. Remote Sens. 7, 1609–1615. doi: 10.1109/JSTARS.2013.2290028

[B37] Hu T.-w. Mao Z. Shi J. Chen W. (2010). The role of taxation in tobacco control and its potential economic impact in china. Tobacco control 19, 58–64. doi: 10.1136/tc.2009.031799 20008158 PMC2921252

[B38] Inoue Y. Sakaiya E. Zhu Y. Takahashi W. (2012). Diagnostic mapping of canopy nitrogen content in rice based on hyperspectral measurements. Remote Sens. Environ. 126, 210–221. doi: 10.1016/j.rse.2012.08.026

[B39] Jia F. Han S. Chang D. Yan H. Xu Y. Song W.. (2020). Monitoring flue-cured tobacco leaf chlorophyll content under different light qualities by hyperspectral reflectance. Am. J. Plant Sci. 11, 1217–1234. doi: 10.4236/ajps.2020.118086

[B40] Jia F. Liu G. Ding S. Yang Y. Fu Y. Wang Z. (2013a). Using leaf spectral reflectance to monitor the effects of shading on nicotine content in tobacco leaves. Ind. Crops Prod. 51, 444–452. doi: 10.1016/j.indcrop.2013.09.027

[B41] Jia F. Liu G. Liu D. Zhang Y. Fan W. Xing X. (2013b). Comparison of different methods for estimating nitrogen concentration in flue-cured tobacco leaves based on hyperspectral reflectance. Field Crops Res. 150, 108–114. doi: 10.1016/j.fcr.2013.06.009

[B42] Jia Kang S. W. (2020). The influence and countermeasures of increasing tobacco tax on cpi and employment. Financial Minds 5, 58–78. doi: 10.20032/j.cnki.cn10-1359/f.2020.05.004

[B43] Jiang J. Johansen K. Stanschewski C. S. Wellman G. Mousa M. A. Fiene G. M.. (2022). Phenotyping a diversity panel of quinoa using uav-retrieved leaf area index, spad-based chlorophyll and a random forest approach. Precis. Agric. 23, 961–983. doi: 10.1007/s11119-021-09870-3

[B44] Johansen K. Morton M. J. Malbeteau Y. M. Aragon B. Al-Mashharawi S. K. Ziliani M. G.. (2019). Unmanned aerial vehicle-based phenotyping using morphometric and spectral analysis can quantify responses of wild tomato plants to salinity stress. Front. Plant Sci. 10, 370. doi: 10.3389/fpls.2019.00370 30984222 PMC6449481

[B45] Johansen K. Morton M. J. Malbeteau Y. Aragon B. Al-Mashharawi S. Ziliani M. G.. (2020). Predicting biomass and yield in a tomato phenotyping experiment using uav imagery and random forest. Front. Artif. Intell. 3, 28. doi: 10.3389/frai.2020.00028 33733147 PMC7861253

[B46] Junying L. Erdeng M. Junjia L. Xiaopeng D. Wenjie D. Xiaohai Z.. (2020a). Method for predicting potassium oxide content in tobacco leaves based on unmanned aerial vehicle (UAV) hyperspectral field, involves generating prediction data of potassium oxide content corresponding to field tobacco leaves. Patent No CN112697724-A.

[B47] Junying L. Erdeng M. Xiaopeng D. Junjia L. Wenjie D. Hua W.. (2020b). Method for predicting total sugar content of tobacco leaves in hyperspectral field based on unmanned aerial vehicle, involves loading coordinate of hyperspectral image data and extracting and processing corresponding original spectral curve. Patent No CN112697725-A.

[B48] Kattenborn T. Leitloff J. Schiefer F. Hinz S. (2021). Review on convolutional neural networks (cnn) in vegetation remote sensing. ISPRS J. Photogramm. Remote Sens. 173, 24–49. doi: 10.1016/j.isprsjprs.2020.12.010

[B49] Ke L. Zhou Q.-b. Wu W.-b. Tian X. Tang H.-j. (2016). Estimating the crop leaf area index using hyperspectral remote sensing. J. Integr. Agric. 15, 475–491. doi: 10.1016/S2095-3119(15)61073-5

[B50] Koonsanit K. Jaruskulchai C. Eiumnoh A. (2012). Band selection for dimension reduction in hyper spectral image using integrated information gain and principal components analysis technique. Int. J. Mach. Learn. Comput. 2, 248. doi: 10.7763/IJMLC.2012.V2.124

[B51] Krezhova D. Dikova B. Maneva S. (2014). Ground based hyperspectral remote sensing for disease detection of tobacco plants. Bulgarian J. Agric. Sci. 20, 1142–1150.

[B52] Latif M. A. (2018). An agricultural perspective on flying sensors: State of the art, challenges, and future directions. IEEE Geosci. Remote Sens. Mag. 6, 10–22. doi: 10.1109/MGRS.2018.2865815

[B53] Li F. (2006). Monitoring tobacco growth and quality base on spectra (Nanjing: Nanjing Agricultural University), 1–154.

[B54] Li Z. Chen Z. Cheng Q. Duan F. Sui R. Huang X.. (2022). Uav-based hyperspectral and ensemble machine learning for predicting yield in winter wheat. Agronomy 12, 202. doi: 10.3390/agronomy12010202

[B55] Li Y. Gu M. Zhang X. Zhang J. Fan H. Li P.. (2014). Engineering a sensitive visual-tracking reporter system for real-time monitoring phosphorus deficiency in tobacco. Plant Biotechnol. J. 12, 674–684. doi: 10.1111/pbi.12171 25187932

[B56] Li Z. Li Z. Fairbairn D. Li N. Xu B. Feng H.. (2019). Multi-luts method for canopy nitrogen density estimation in winter wheat by field and uav hyperspectral. Comput. Electron. Agric. 162, 174–182. doi: 10.1016/j.compag.2019.04.005

[B57] Liang L. Di L. Huang T. Wang J. Lin L. Wang L.. (2018). Estimation of leaf nitrogen content in wheat using new hyperspectral indices and a random forest regression algorithm. Remote Sens. 10, 1940. doi: 10.3390/rs10121940

[B58] Liang T. Wang J. Zhang Y. Xi J. Zhou H. Wang B.. (2014). “Spectral characteristics of tobacco cultivars with different nitrogen efficiency and its relationship with nitrogen use,” in International Conference on Computer and Computing Technologies in Agriculture. (Beijing, China: Springer), 239–246. doi: 10.1007/978-3-642-54341-8_25

[B59] Liao X. Yue H. Liu R. Luo X. Luo B. Lu M.. (2020). Launching an unmanned aerial vehicle remote sensing data carrier: concept, key components and prospects. Int. J. Digit. Earth 13, 1172–1185. doi: 10.1080/17538947.2019.1698664

[B60] Lin J. Chen Y. Pan R. Cao T. Cai J. Yu D.. (2022). Camffnet: A novel convolutional neural network model for tobacco disease image recognition. Comput. Electron. Agric. 202, 107390. doi: 10.1016/j.compag.2022.107390

[B61] Liu W. Li Q. (2017). An efficient elastic net with regression coefficients method for variable selection of spectrum data. PloS One 12, 1–13. doi: 10.1371/journal.pone.0171122 PMC5289531 28152003

[B62] Liu S. Shi Q. (2020). Multitask deep learning with spectral knowledge for hyperspectral image classification. IEEE Geosci. Remote Sens. Lett. 17, 2110–2114. doi: 10.1109/LGRS.2019.2962768

[B63] Liu T. Xu T. Yu F. Yuan Q. Guo Z. Xu B. (2021). A method combining elm and plsr (elm-p) for estimating chlorophyll content in rice with feature bands extracted by an improved ant colony optimization algorithm. Comput. Electron. Agric. 186, 106177. doi: 10.1016/j.compag.2021.106177

[B64] Long Z. Xiaoyu M. Zhigang L. Yong L. (2019). “Application of hyperspectral imaging technology in classification of tobacco leaves and impurities,” in 2019 2nd International Conference on Safety Produce Informatization (IICSPI). (Chongqing, China: IEEE), 157–160. doi: 10.1109/IICSPI48186.2019.9095975

[B65] Ma J. Zheng B. He Y. (2022). Applications of a hyperspectral imaging system used to estimate wheat grain protein: A review. Front. Plant Sci. 13, 837200. doi: 10.3389/fpls.2022.837200 35463397 PMC9024351

[B66] Maes W. H. Steppe K. (2019). Perspectives for remote sensing with unmanned aerial vehicles in precision agriculture. Trends Plant Sci. 24, 152–164. doi: 10.1016/j.tplants.2018.11.007 30558964

[B67] Mahlein A.-K. Kuska M. T. Behmann J. Polder G. Walter A. (2018). Hyperspectral sensors and imaging technologies in phytopathology: State of the art. Annu. Rev. Phytopathol. 56, 535–558. doi: 10.1146/annurev-phyto-080417-050100 30149790

[B68] Meacham-Hensold K. Montes C. M. Wu J. Guan K. Fu P. Ainsworth E. A.. (2019). High-throughput field phenotyping using hyperspectral reflectance and partial least squares regression (plsr) reveals genetic modifications to photosynthetic capacity. Remote Sens. Environ. 231, 111176. doi: 10.1016/j.rse.2019.04.029 31534277 PMC6737918

[B69] Moghadam P. Ward D. Goan E. Jayawardena S. Sikka P. Hernandez E. (2017). “Plant disease detection using hyperspectral imaging,” in 2017 International Conference on Digital Image Computing: Techniques and Applications (DICTA). (Sydney, NSW, Australia: IEEE), 1–8. doi: 10.1109/DICTA.2017.8227476

[B70] Mountrakis G. Im J. Ogole C. (2011). Support vector machines in remote sensing: A review. ISPRS J. Photogramm. Remote Sens. 66, 247–259. doi: 10.1016/j.isprsjprs.2010.11.001

[B71] Mulla D. J. (2013). Twenty five years of remote sensing in precision agriculture: Key advances and remaining knowledge gaps. Biosyst. Eng. 114, 358–371. doi: 10.1016/j.biosystemseng.2012.08.009

[B72] Ort D. R. Merchant S. S. Alric J. Barkan A. Blankenship R. E. Bock R.. (2015). Redesigning photosynthesis to sustainably meet global food and bioenergy demand. Proc. Natl. Acad. Sci. 112, 8529–8536. doi: 10.1073/pnas.1424031112 26124102 PMC4507207

[B73] Park B. Lu R. (2015). Hyperspectral imaging technology in food and agriculture (New York: Springer). doi: 10.1007/978-1-4939-2836-1

[B74] Peng Y. Gitelson A. A. (2012). Remote estimation of gross primary productivity in soybean and maize based on total crop chlorophyll content. Remote Sens. Environ. 117, 440–448. doi: 10.1016/j.rse.2011.10.021

[B75] Pu R. (2017). Hyperspectral remote sensing: fundamentals and practices (Boca Raton, Florida: CRC Press). doi: 10.1201/9781315120607

[B76] Qiao H. Mei W. Yang Y. Yong W. Zhang J. Hua Y. (2011). “Study on relationship between tobacco canopy spectra and lai,” in International Conference on Computer and Computing Technologies in Agriculture. (Nanchang, China: Springer), 650–657. doi: 10.1007/978-3-642-18336-2_79

[B77] Qu Y. Jiao S. (2018). Quantitative estimation of tobacco copper ion content from hyperspectral data by inverting a modified radiative transfer model: Algorithm and preliminary validation. J. Spectrosc. 2018, 1–12. doi: 10.1155/2018/8508737

[B78] Rivera J. P. Verrelst J. Gómez-Dans J. Muñoz-Marí J. Moreno J. Camps-Valls G. (2015). An emulator toolbox to approximate radiative transfer models with statistical learning. Remote Sens. 7, 9347–9370. doi: 10.3390/rs70709347

[B79] Sahu A. Dante H. (2018). Non-destructive rapid quality control method for tobacco grading using visible near-infrared hyperspectral imaging. In Image Sens. Technol.: Mater. Devices Sys. Appl. V (SPIE) 10656, 1065603. doi: 10.1117/12.2305091

[B80] Shen H. Jiang J. Tang Z. Liu B. Tong Y. (2017). Study on relationship between the main nitrogen compounds and sensory quality in flue-cured tobacco leaves. J. Yunnan Agric. Univ. 32, 558–563. doi: 10.16211/j.issn.1004-390X(n).2017.03.023

[B81] Soares F. L. Marcelo M. C. Porte L. M. Pontes O. F. Kaiser S. (2019). Inline simultaneous quantitation of tobacco chemical composition by infrared hyperspectral image associated with chemometrics. Microchem. J. 151, 104225. doi: 10.1016/j.microc.2019.104225

[B82] Sothe C. De Almeida C. Schimalski M. La Rosa L. Castro J. Feitosa R.. (2020). Comparative performance of convolutional neural network, weighted and conventional support vector machine and random forest for classifying tree species using hyperspectral and photogrammetric data. GISci. Remote Sens. 57, 369–394. doi: 10.1080/15481603.2020.1712102

[B83] Sun W. Du Q. (2019). Hyperspectral band selection: A review. IEEE Geosci. Remote Sens. Mag. 7, 118–139. doi: 10.1109/MGRS.2019.2911100

[B84] Sun J. Wu X. Zhang X. Li Q.. (2016) Identification of moisture content in tobacco plant leaves using outlier sample eliminating algorithms and hyperspectral data. Biochem. Biophys. Res. Commun. 471, 226–232. doi: 10.1016/j.bbrc.2016.01.125 26809097

[B85] Svotwa E. Masuka A. J. Maasdorp B. Murwira A. Shamudzarira M. (2013). Remote sensing applications in tobacco yield estimation and the recommended research in zimbabwe. Int. Scholarly Res. Notices 2013, 1–7. doi: 10.1155/2013/941873

[B86] Teke M. Deveci H. S. Haliloğlu O. Gürbüz S. Z. Sakarya U. (2013). “A short survey of hyperspectral remote sensing applications in agriculture,” in 2013 6th international conference on recent advances in space technologies (RAST). (Istanbul, Turkey: IEEE), 171–176. doi: 10.1109/RAST.2013.6581194

[B87] Vane G. Goetz A. F. (1993). Terrestrial imaging spectrometry: Current status, future trends. Remote Sens. Environ. 44, 117–126. doi: 10.1016/0034-4257(93)90011-L

[B88] Verrelst J. Malenovský Z. van der Tol C. Camps-Valls G. Gastellu-Etchegorry J.-P. Lewis P.. (2019). Quantifying vegetation biophysical variables from imaging spectroscopy data: A review on retrieval methods. Surv. Geophys. 40, 589–629. doi: 10.1007/s10712-018-9478-y 36081834 PMC7613341

[B89] Vibhute A. D. Kale K. Dhumal R. K. Mehrotra S. (2015). “Hyperspectral imaging data atmospheric correction challenges and solutions using quac and flaash algorithms,” in 2015 International Conference on Man and Machine Interfacing (MAMI). (Bhubaneswar, India: IEEE), 1–6. doi: 10.1109/MAMI.2015.7456604

[B90] Wagner F. H. Sanchez A. Tarabalka Y. Lotte R. G. Ferreira M. P. Aidar M. P.. (2019). Using the u-net convolutional network to map forest types and disturbance in the atlantic rainforest with very high resolution images. Remote Sens. Ecol. Conserv. 5, 360–375. doi: 10.1002/rse2.111

[B91] Wan L. Zhou W. He Y. Wanger T. C. Cen H. (2022). Combining transfer learning and hyperspectral reflectance analysis to assess leaf nitrogen concentration across different plant species datasets. Remote Sens. Environ. 269, 112826. doi: 10.1016/j.rse.2021.112826

[B92] Wang C. Chen Q. Fan H. Yao C. Sun X. Chan J.. (2021). Evaluating satellite hyperspectral (orbita) and multispectral (landsat 8 and sentinel-2) imagery for identifying cotton acreage. Int. J. Remote Sens. 42, 4042–4063. doi: 10.1080/01431161.2021.1887543

[B93] Wang M. Li X. J. Lu Y. Y. Guo S. L. (2011). Tobacco pest monitoring feasibility analysis based on rs. Adv. Mater. Res. (Trans Tech Publ) 217, 1516–1519. doi: 10.4028/www.scientific.net/AMR.217-218.1516

[B94] Wang M. Li X.-j. Yao Q.-q. Liu Y. (2012). Extraction of diseases and insect pests for tobacco based on hyperspectral remote sensing. Geodetski list 66, 209–216.

[B95] Wang Z. Skidmore A. K. Darvishzadeh R. Wang T. (2018). Mapping forest canopy nitrogen content by inversion of coupled leaf-canopy radiative transfer models from airborne hyperspectral imagery. Agric. For. meteorol. 253, 247–260. doi: 10.1016/j.agrformet.2018.02.010

[B96] Watts A. C. Ambrosia V. G. Hinkley E. A. (2012). Unmanned aircraft systems in remote sensing and scientific research: Classification and considerations of use. Remote Sens. 4, 1671–1692. doi: 10.3390/rs4061671

[B97] Weiss M. Jacob F. Duveiller G. (2020). Remote sensing for agricultural applications: A meta-review. Remote Sens. Environ. 236, 111402. doi: 10.1016/j.rse.2019.111402

[B98] Xiang T.-Z. Xia G.-S. Zhang L. (2019). Mini-unmanned aerial vehicle-based remote sensing: techniques, applications, and prospects. IEEE Geosci. Remote Sens. Mag. 7, 29–63. doi: 10.1109/MGRS.2019.2918840

[B99] Xin-Zhong W. Guo-Shun L. Hong-Chao H. Zhen-Hai W. Qing-Hua L. Xu-Feng L.. (2009). Determination of management zones for a tobacco field based on soil fertility. Comput. Electron. Agric. 65, 168–175. doi: 10.1016/j.compag.2008.08.008

[B100] Yu K. Fang S. Zhao Y. (2021). Heavy metal hg stress detection in tobacco plant using hyperspectral sensing and data-driven machine learning methods. Spectro. Acta Part A: Mol. Biomol. Spectrosc. 245, 118917. doi: 10.1016/j.saa.2020.118917 32949945

[B101] Yuan H. Yang G. Li C. Wang Y. Liu J. Yu H.. (2017). Retrieving soybean leaf area index from unmanned aerial vehicle hyperspectral remote sensing: Analysis of rf, ann, and svm regression models. Remote Sens. 9, 309. doi: 10.3390/rs9040309

[B102] Yusuf B. L. He Y. (2011). Application of hyperspectral imaging sensor to differentiate between the moisture and reflectance of healthy and infected tobacco leaves. Afr. J. Agric. Res. 6, 6267–6280. doi: 10.5897/AJAR11.1281

[B103] Zhang Y. Hui J. Qin Q. Sun Y. Zhang T. Sun H.. (2021). Transfer-learning-based approach for leaf chlorophyll content estimation of winter wheat from hyperspectral data. Remote Sens. Environ. 267, 112724. doi: 10.1016/j.rse.2021.112724

[B104] Zhang M. Ma J. Gong M. (2017). Unsupervised hyperspectral band selection by fuzzy clustering with particle swarm optimization. IEEE Geosci. Remote Sens. Lett. 14, 773–777. doi: 10.1109/LGRS.2017.2681118

[B105] Zhao J. Zhong Y. Jia T. Wang X. Xu Y. Shu H.. (2018). Spectral-spatial classification of hyperspectral imagery with cooperative game. ISPRS J. Photogramm. Remote Sens. 135, 31–42. doi: 10.1016/j.isprsjprs.2017.10.006

[B106] ZhengYang Z. XinMing M. GuoShun L. FangFang J. HongBo Q. YingWu Z.. (2011). A study on hyperspectral estimating models of tobacco leaf area index. Afr. J. Agric. Res. 6, 289–295. doi: 10.5897/AJAR10.533

[B107] Zhong Y. Wang X. Xu Y. Wang S. Jia T. Hu X.. (2018). Mini-uav-borne hyperspectral remote sensing: From observation and processing to applications. IEEE Geosci. Remote Sens. Mag. 6, 46–62. doi: 10.1109/MGRS.2018.2867592

[B108] Zhu H. Chu B. Zhang C. Liu F. Jiang L. He Y. (2017). Hyperspectral imaging for presymptomatic detection of tobacco disease with successive projections algorithm and machine-learning classifiers. Sci. Rep. 7, 1–12. doi: 10.1038/s41598-017-04501-2 28646177 PMC5482814

[B109] Zhu W. Sun Z. Yang T. Li J. Peng J. Zhu K.. (2020). Estimating leaf chlorophyll content of crops *via* optimal unmanned aerial vehicle hyperspectral data at multi-scales. Comput. Electron. Agric. 178, 105786. doi: 10.1016/j.compag.2020.105786

